# Non-uniform chromosomal SNP density biases sites of meiotic crossovers in *Drosophila melanogaster*

**DOI:** 10.1093/g3journal/jkag095

**Published:** 2026-04-15

**Authors:** Savanna Hinson, Ryan Sangston, Karol Cichewicz, Guruprasad Konduru, Ishan Parikh, Jay Hirsh

**Affiliations:** Department of Biology, University of Virginia, 485 McCormick Rd, Charlottesville, VA 22903, United States; Department of Biology, University of Virginia, 485 McCormick Rd, Charlottesville, VA 22903, United States; Department of Biology, University of Virginia, 485 McCormick Rd, Charlottesville, VA 22903, United States; Bioinformatics Core, University of Virginia, 1312 Pinn Hall, Charlottesville, VA 22908, United States; Department of Biology, University of Virginia, 485 McCormick Rd, Charlottesville, VA 22903, United States; Department of Biology, University of Virginia, 485 McCormick Rd, Charlottesville, VA 22903, United States

**Keywords:** recombination, genetic mapping, dopamine

## Abstract

Here we localize a genetic suppressor that enhances the reduced locomotor activity phenotype of flies lacking brain dopamine. We utilized a bulk segregant analysis mapping strategy coupled with whole genome sequencing, mapping the trait to a roughly 3.5 mega-base region of the X chromosome. However, this mapping yielded ∼5-fold lower resolution than anticipated, due to an uneven distribution of single nucleotide polymorphisms (SNPs) between the X chromosomes of the 2 recombining lines. This uneven SNP distribution was associated with recombination events biased toward regions of low SNP density, and away from the more SNP dense regions associating with the activity phenotype. We find that nearly perfect mapping of X chromosome visible markers occurs only in historical data from a time before the establishment of discrete genetic background strains. This suggests that genetic uniformity in early *Drosophila* studies may have contributed to more consistent recombination frequencies, whereas modern mapping efforts are complicated by variability in SNP distribution across recombining strains. These findings highlight challenges in *Drosophila* genetic mapping in situations where altered SNP density can skew recombination, complicating trait localization.

## Introduction

Genetic mapping enables the identification of loci associated with phenotypic traits, and the expected resolution of genetic mapping relies on the simplified assumption that meiotic crossovers (COs) are randomly distributed across the genome. However, deviations from this assumption have been observed in many species, including *Drosophila melanogaster*. Studies in *Drosophila* have shown variation in recombination rates across chromosomes, with regions near centromeres and telomeres suppressing COs and significant variation also within the interior of a chromosome (Lindsley and Sandler 1977; Chan et al. 2012; Comeron et al. 2012; Hughes et al. 2018).

Here, we examine how variations in parental single nucleotide polymorphism (SNP) density alter CO frequency and locations in recombinant offspring. Our work began with an investigation into the genetic basis of locomotor recovery in dopamine (DA)-deficient flies. DA has critical functions in both the central nervous system (CNS) and in vital cuticular processes in *Drosophila*. The biosynthesis of DA is regulated by tyrosine hydroxylase (TH), encoded by the *pale* (*ple*) gene, which generates distinct splice variants for CNS and cuticular functions (Birman et al. 1994). By restoring cuticular DA synthesis in a *ple*-lethal background, we generated viable flies that are entirely devoid of CNS DA (Riemensperger et al. 2011; Cichewicz et al. 2017). These flies initially exhibited reduced locomotor activity, consistent with previous findings showing that DA is required for normal behaviors (Yellman et al. 1997; Friggi-Grelin et al. 2003; Lima and Miesenböck 2005). While “cleaning up” the genetic background in this line, we discovered a genetic suppressor of the hypoactivity in these flies, leading to near normal levels of locomotion, with no restoration of DA.

To identify the genetic factors responsible for this unexpected recovery of locomotor function, we employ a bulk segregant analysis (BSA) mapping strategy in which recombinant flies were sequenced individually using whole genome sequencing (WGS) and then grouped into high and low activity groups for association testing. Our analysis maps the trait to a 3.5 mega-base (Mb) region on the X chromosome, a substantially lower resolution than expected. We identify an uneven distribution of SNPs within the X chromosomes of the two parental lines, which is associated with a bias in CO location and frequency. Regions of near homozygosity, or low SNP density, exhibit higher CO frequencies, while regions with higher SNP density show reduced COs.

## Materials and methods

### Starting brain DA-deficient flies

Flies were as per Cichewicz et al (2017). These flies have a 3rd chromosome bacterial artificial chromosome (BAC) containing a *pale* gene modified to rescue in epidermis but not CNS, which was then recombined into a *ple* lethal background. The stock was maintained containing a floating 3rd chromosome balancer, TM6b.

### Measurement of locomotor activity

Locomotor activity was measured using the Drosophila Activity Monitor System (DAM, Trikinetics, MA, USA) at 22C, 60% RH. Age-matched flies were loaded into 3 mm ID glass tubes with standard cornmeal food on one end, and a cotton plug on the other. Entrainment was in a 12:12 light/dark cycle for 5 d, at which point they were switched to constant darkness, and activity was measured for an additional 5 d.

### Feeding of 3IY and methamphetamine

3-Iodo-L-tyrosine (3-IY) (I8250, Sigma) and (+) methamphetamine HCL (M8750, Sigma) were mixed directly into melted fly food at final concentrations of 1.25 and 1 mg/ml, respectively, was dissolved in 1 ml of water at the 10× final concentration and mixed with 9 ml of melted fly food. The Trikinetics activity monitor tubes were loaded with a plug of food at one end, with drug feeding throughout the course of the experiment.

### HPLC analysis of DA and serotonin

Conditions were as per (Hardie and Hirsh 2006), with a mobile phase consisting of 12% acetonitrile, 0.4 mM decanesulfonic acid, 50 mM sodium citrate/acetate, pH 4.5, 0.1 mM ethylenediaminetetraacetic acid.

### Interbreeding *DD-Hi and DD-Lo* for bulk segregant analysis

*DD-Hi* and *DD-Lo* flies were allowed to freely intermate for 15 generations. The study started with group of ∼80 *DD-Hi* females and ∼55 *DD-Lo* males that were allowed to mate and lay eggs for 5 d in Tupperware containers with standard cornmeal media and yeast, at 22 °C, 60% RH. Parental lines were cleared as soon as progeny eclosed. Progeny were transferred to fresh Tupperware containers to continue interbreeding with a transferred population of several hundred. After 15 generations, 788 males were collected for assay of locomotor activity as above. The 15-generation recombinant male flies were separated into 2 bulked groups of 45 flies each, a low activity group with activity levels from 95 to 238 counts/d, mean = 194, and a high activity group with 1,460 to 1,861 counts/d, mean = 1,609. DNA from individual flies was isolated for sequencing. Additionally, *DD-Hi* and 3 *DD-Lo* females were sequenced for analysis of parentage.

### Sequencing

Libraries were constructed for paired-end Illumina sequencing in a HiSeq X. DNA isolation from whole flies was performed using an alcohol precipitation protocol adapted from Bergland et al. (2014). Fragmentation, end repair, dA-tailing, and size selection were then achieved using the NEBNext Ultra II FS DNA Library Prep Kit for Illumina (NE Biolabs, Beverly, MA, USA). Indexing primers for multiplexing were subsequently ligated using NEBNext Multiplex Oligos for Illumina (Dual Index Primers Set 1) (NE Biolabs, Beverly, MA, USA). We performed paired-end sequencing, generating approximately 4 to 6 million reads per sample with a read length of 150 bp, and removed adapter contamination using fastp. Of the 45 recombinant male flies sequenced for each group, 40 high activity recombinant and 44 low activity recombinant sequences passed quality control and were used for downstream analysis. Of the 3 *DD-Hi* and 3 *DD-Lo* parental female flies sequenced, 3 *DD-Hi* and 2 *DD-Lo* sequences passed quality control and were used for downstream lineage analysis.

### SNP calling

SNPs were called using a combination of bioinformatic tools using a standard variant discovery pipeline consisting of adaptor trimming, alignment to the *Drosophila* reference genome, duplicate removal, and variant calling using GATK.

fastp was used in paired end mode and input 5′-AGATCGGAAGAGCACACGTCTGAACTCCAGTCA-3 as the adapter sequence for read 1 and 5′-GATCGGAAGAGCGTCGTGTAGGGAAAGAGTGT-3′ as the adapter sequence for read 2 (Chen et al. 2018). Default fastp flags for quality control and trimming were used.

Alignment to the *Drosophila* genome assembly release 6 plus ISO1 MT (GCF_000001215.4) was performed using the BWA mem algorithm in paired end mode (Li and Durbin 2009). Alignment flags included -M for downstream Picard compatibility and -a to allow for all output alignments. BAM files were sorted by read name using samtools for duplicates to be marked in the next step (Danecek et al. 2021). Duplicate reads were removed using MarkDuplicates from Broad Institute's Picard toolkit (https://broadinstitute.github.io/picard/) flagging REMOVE_DUPLICATES as true. BAM files were then coordinate sorted using samtools, which is required for downstream analysis. Following alignment and duplicate removal, the Germline short variant discovery (SNPs+Indels) pipeline was followed using the Broad Institute's Genome Analysis Toolkit (GATK) (https://gatk.broadinstitute.org/hc/en-us). The following GATK commands were used: HaplotypeCaller called variants on each sample's BAM file and used the flag -ERC GVCF for reference model calling, which is necessary for multi-sample variant calling; GenomicsDBImport imported multiple gVCF files into a GenomicsDB workspace; and GenotypeGVCF called genotypes of gVCF files from a database that included parental data. GATK variant calling for 84 samples used –sample-ploidy 1 and 5 parental samples used –sample-ploidy 2.

In cases where SNPs are not detected at given base locations, it is inferred that these base pairs share the same origin as the nearest base pair for which a SNP was successfully called and matched to a parental genotype.

The starting *DD-Hi* and *DD-Lo* stocks showed a high level of isogeny on the X chromosome, with 99.93% homozygosity for *DD-Hi* and 99.95% for *DD-Lo*. Regions of the *DD-Hi* and *DD-Lo* parental chromosomes that contained variation among samples were removed from our analysis (7.9% and 5.8% of SNPs were heterozygous, respectively). We focused solely on homozygous SNPs within each parental line to eliminate any confusion over non-fixed SNPs.

### Association analysis

The variant call file generated from the GATK pipeline (https://gatk.broadinstitute.org/hc/en-us) was analyzed for statistical association using PLINK v1.9.0-b.7.8 (Chang et al. 2015). For association analysis, we removed parental samples from combined vcf file and generated vcf file containing the only 84 male samples. We used the following arguments as inputs to PLINK along with the phenotype file and the 84 male samples vcf file as inputs.

–assoc fisher –allow-extra-chr –allow-no-sex –pheno phenotype_file1.txt

*P* values were generated using the Fisher's exact test option. Manhattan plots were generated in R using ggplot2 (Wickham 2016).

### Lineage analysis

Parental identity was identified using the vcf generated from Broad Institute's GATK pipeline. Each sample was evaluated at each SNP to determine if it matched uniquely to a parental line. SNPs for which a given sample had no supporting reads were ignored. SNPs matching any of the 3 parental samples were assigned a source lineage only if the genotype in the parental samples differed between *DD-Hi* and *DD-Lo*. Next, each sample was evaluated using the locations for which we had explicit sequencing data. Base pair locations which were missing sequencing data were interpolated to be the same as the surrounding SNPs. Ambiguous regions, which contain non-informative regions of the chromosome or missing sequencing reads, are indicated as white in Fig. 4.

### Determining fraction of *DD-Hi* parentage

*DD-Hi* parentage was estimated within each SNP dense region by assigning either 0 for a full *DD-Lo* tract, 1 for a full *DD-Hi* tract, or a fraction representing the amount of *DD-Hi* for each fly. The mean values for high and low activity recombinants in each region were plotted using ggplot2 in R with the distribution of parentage values shown by the violin plots (Wickham 2016). *P*-values were generated using a Wilcoxon Rank Sum test followed by Bonferroni correction for multiple comparisons.

### Estimating recombination frequency

Recombination frequency was estimated by counting changes in parental identity within the SNP dense regions, or between the smallest SNP sparse region (Fig. 5). The total number of COs for a given region was divided by the length of the region to normalize to numbers of COs per 1 Mb. This was divided by 84 (total number of flies) and then 5 (total number of generations with recombination occurring) to quantify the recombination frequencies for a single meiosis within in each region. Bootstrap resampling (*n* = 10,000) was then used to generate 95% confidence intervals for each region. Statistical significance of the difference in CO rate between SNP dense and SNP sparse regions was assessed using an exact conditional Poisson rate test, in which the observed count in each dense region is treated as binomial given total COs across both regions under the null hypothesis of equal per Mb rates.

### Long read sequencing to detect X chromosome inversions

Nanopore long read sequencing was used to search for inversions, with sequence coverage of 23.4× for *DD-Lo* and 17.5× for *DD-Hi,* respectively. Structural variant analysis was performed using SVIM, which outputs all possible structural variants, even ones that have minimal read support. SVIM outputted no inversions with strong support. *DD-Lo* was found to have at most 29 inversions with very weak support with low numbers of supporting reads in the single digits, and at most 20 inversions with very weak support in *DD-Hi.* Only 0.41% and 0.43% of the X chromosome has 0 coverage for DD-Lo and DD-Hi, respectively, as measured by samtools depth. Therefore, we conclude that there are no X chromosome inversions inhibiting recombination in our fly strains.

## Results

### DA-deficient flies recover normal locomotor activity independent of neural DA

**Fig. 1.**
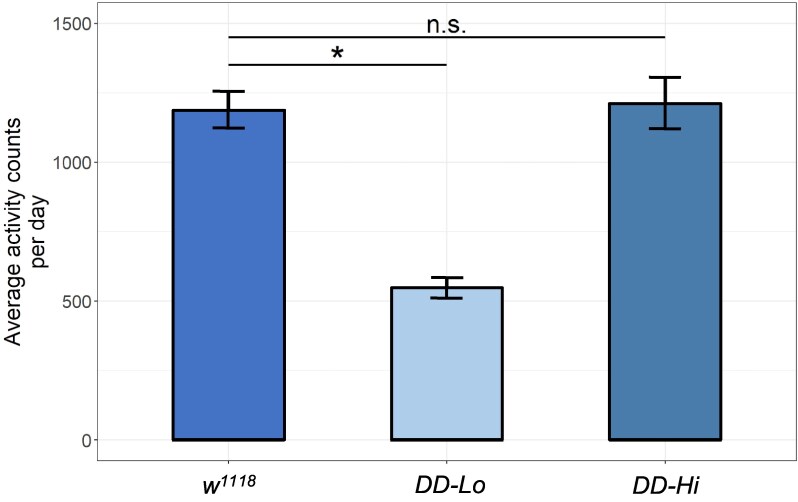
Activity levels of the flies used in this study. Average activity levels in constant darkness in our lab stock of *w^1118^*, *DD-Lo* and *DD-Hi*. 3 to 5-d old adult flies were entrained to 12:12 LD cycles for 5 d, then switched to constant darkness (DD). Activity levels were averaged over 3 d in DD. Significance indicates *P* < 0.05 tested by an ANOVA followed by Tukey's honest test of significance. *n* = 64 per condition.

A viable line of flies wholly lacking in brain DA was isolated by Cichewicz et al (2017), by rescuing a lethal mutation in the *pale (ple)* gene with a 3rd chromosome BAC that selectively rescues *ple^+^* function in the hypoderm, but not in CNS. As expected, these flies show reduced levels of locomotor activity relative to wild type controls. In the process of “cleaning up” the genetic background of these flies, they were crossed to our laboratory stock of *w^1118^*, and the relevant 3rd chromosomes were reisolated by single pair matings. To our surprise, a small number of the stocks resulting from the single pair matings show normal levels of locomotor activity. Activity levels of one of these stocks, which we name *DD-Hi*, are shown in Fig. 1, compared to our lab *w^1118^* and to stocks showing the more expected and reduced levels of activity, hereafter named *DD-Lo*.

**Fig. 2.**
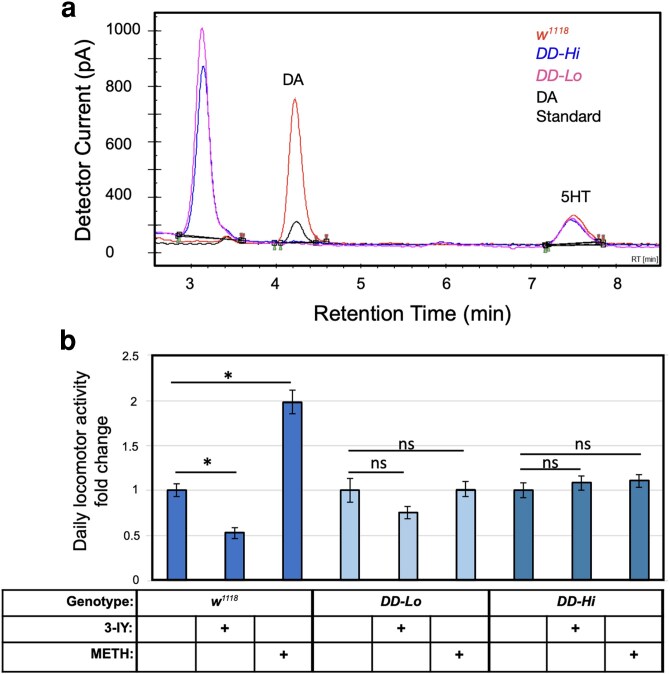
Dopamine deficient lines lack DA by HPLC or via pharmacologic intervention. a) Neither *DD-Lo* or *DD-Hi* brain extracts show detectable DA. A peak with the retention time characteristic of DA was detected in the standard and in brain extracts from *w^1118,^* but no DA peak was detected in brains of *DD-Lo* or *DD-Hi.* Normal levels of 5-HT were detected in all 3. The peak with the retention time of 3.1 min is associated with red eye pigment and was detected in the 2 DA-deficient genotypes, which express the *white^+^* transgene. Y axis scale: 1V = 2 nA detector current. b) Locomotor activity analysis of flies treated with the TH inhibitor 3-iodotyrosine (3-IY), and methamphetamine (METH), demonstrates a DA-independent mechanism responsible for hyperactivity of *DD-Hi* line. Locomotor activity was normalized to No Drug control for each genotype. [*P < 5E−04, one-way ANOVA (Bonferroni-corrected), error bars represent SEM, *n* = 64 for each group.

Given the surprisingly high levels of locomotor activity levels in the *DD-Hi* line, we sought to validate that these flies were still deficient in brain DA. We did this using two approaches, first measuring DA in brain extracts by HPLC with electrochemical detection (Hardie and Hirsh 2006), and second, by probing these flies pharmacologically. Both of the *DD* lines show no detectable DA by HPLC analysis, whereas serotonin levels are unaffected (Fig. 2a). Since this approach could miss trace levels of brain DA, we followed up with a pharmacologic approach. Cichewicz et al. (2017) found that locomotor activity of the initial stock of brain DA deficient flies was insensitive to feeding of the TH inhibitor, 3-IY. The *DD-Lo* and *DD-Hi* lines are similarly insensitive to 3-IY, whereas the *w^1118^* control line shows reduced locomotor activity after 3-IY feeding (Fig. 2b). In addition, we fed the flies methamphetamine, which stimulates DA release and blocks reuptake (Nestler 2004), thus stimulating locomotor activity in adult *Drosophila* (Andretic et al. 2005). The control *w^1118^* line shows greatly enhanced locomotion after methamphetamine feeding, but the locomotor activity of the *DD-Hi* or *DD-Lo* lines is unaffected (Fig. 2b). These results indicate that the *DD-Hi* flies have regained normal levels of locomotor activity in a manner that is independent of even trace levels of neural DA.

### Identification of the gene(s) responsible for the altered locomotor activity in *DD-Lo* vs *DD-Hi*

We sought to identify the genetic element(s) responsible for the unexpected *DD-Hi* phenotype via BSA with WGS (Michelmore et al. 1991). In this study, the *DD-Hi* and *DD-Lo* stocks were allowed to freely intermate for fifteen generations, followed by measurement of locomotor activity in individual recombinant males, then identifying the SNPs that associate significantly with activity levels.

**Fig. 3.**
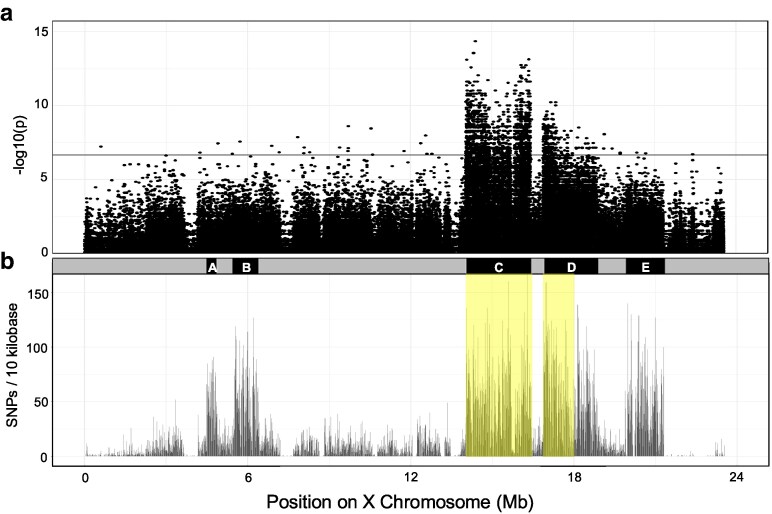
A bulk segregant analysis strategy localizes the SNPs associating with high vs low locomotor activity after 15 generations free recombination between the *DD-Hi* and *DD-Lo* lines, and *DD-Hi* and *DD-Lo* X chromosomes show unevenly distributed single nucleotide differences. a) Significantly associating SNPs were determined as *P* < 0.05 after Bonferroni correction, as shown by the black horizontal line. b) Non-uniform distribution of X chromosome SNPs between the *DD-Lo* and *DD-Hi* X chromosomes. SNP density is plotted as SNPs/kb, with SNPs determined by GATK. The average number of SNPs within the SNP dense regions/10 kb are 23 for *DD-Lo* and 27 for *DD-Hi.* The regions that show association with the *DD-Hi/Lo* bulked phenotypic differences from the 15-generation BSA are shown highlighted in yellow. These regions are exclusively within the regions of enhanced SNP density. The SNP dense regions are labeled A through E in black at the top of the figure. [*n* = 40 high activity and 44 low activity recombinants]. Regions A-E have lengths of 0.4, 0.8, 2.6, 1.9, and 1.5 Mb, respectively.

Association was performed by measuring activity levels in ∼800 recombinant male flies (see Methods), then dividing these into pools representing the high and low extremes of locomotor activity levels, then performing WGS on each fly. We generated 4 to 6 million sequencing reads per fly, with 44 low locomotor activity recombinant sequences and 40 high activity sequences. SNPs were identified as in Methods, with SNPs showing significant association with the locomotor activity pools on the X chromosome mapping to a ∼3.5 Mb region of the X chromosome from 14 to 18 Mb, with a gap at ∼16.5–17 Mb (Fig. 3a). This was substantially lower resolution than we had hoped for. Since recombination occurs only in female *Drosophila*, and females contain 2 X chromosomes vs the single X chromosome in males, a given X chromosome will undergo on average 10 generations of meiosis in females, and given that only half of the chromatids will have one CO in each meiosis, there will be 5 opportunities for single reciprocal recombination events (Jones et al. 2024).

This result was disappointing, since if there is on average one CO per meiosis per X chromosome (Miller et al. 2012) and these were randomly distributed, we should have achieved far better resolution, with one CO on average every 0.74 Mb (23.5 Mb/2^5^). We ignored the autosomes since (i) the high activity phenotype occurs only in males and mapped predominantly to the X chromosome and (ii) the difficulty in analyzing diploid 2nd and 3rd chromosomes where it is not possible to determine individual haplotypes.

There are several possible explanations for this failure to tightly resolve the SNPs associating with the locomotor activity differences between *DD-Hi* and *DD-Lo*. First, an X chromosome inversion could be suppressing local recombination frequency. Second, there could be a bias in where recombination is occurring, due to an uneven distribution of SNPs across the X chromosome. Third, the entire associating region may be required for the altered locomotor activity between *DD-Hi* and *DD-Lo* phenotypes, with many loci acting additively. We investigated each of these possibilities.

### Could inversions be affecting X chromosome recombination frequency?

The presence of chromosomal inversions could be an additional factor suppressing homologous recombination [reviewed by Hoffmann and Rieseberg (2008)]. We sequenced *DD-Hi* and *DD-Lo* using long read Nanopore sequencing (Methods), but no inversions were found that were strongly supported by the data.

### Non-uniform SNP distribution along the X chromosome

To investigate the possibility that the sequence divergence between the parental X chromosomes could be biasing sites of recombination, we examined the distribution of single nucleotide differences in the *DD-Hi* vs *DD-Lo* parental lines. We find an uneven pattern of SNPs in both *DD-Hi* and *DD-Lo* parental X chromosomes that are clustered into 5 discrete regions averaging 25 SNPs/10 kb, or 0.25% vs 4 SNPs/10 kb in the SNP sparse regions, or 0.04% (Fig. 3b). It is of interest that 2 of these SNP dense regions overlap with the regions associated with the differences in locomotor activity between *DD-Hi* and *DD-Lo* identified in the mapping analysis (Fig. 3). Since homologous pairing is a general prerequisite for recombination (Mao et al. 2008), this uneven distribution of SNPs may bias recombination break points to the more highly conserved, SNP sparse regions.

**Fig. 4.**
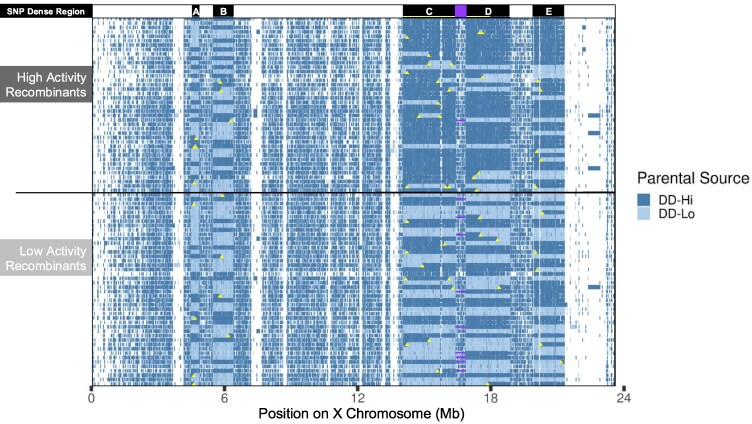
Single nucleotide differences of each individual recombinant fly from the bulk segregant analysis. Each horizontal line shows a sequenced individual fly, with activity levels ordered by activity from highest activity at the top to lowest at the bottom, with the horizontal line in the center dividing the high vs low bulked flies. Informative SNPs were used to determine if a region matched *DD-Hi* (dark blue), *DD-Lo* (light blue), reference (white), unknown, or possessed a unique (gray) SNP. The SNP dense regions a–e are shown in black at the top of the figure, with the yellow bar in regions c and d indicating the regions showing significant association in the BSA. Reciprocal recombination events within the SNP dense regions are determined by a switch in parentage from *DD-Hi* to *DD-Lo,* or vice versa, and are indicated by a yellow triangle. The smallest SNP sparse region is indicated with a purple box at the top of the figure. This region spans 0.4 Mb and contains a minimum of 10 reciprocal crossover events. Regions a–e contain 8, 7, 23, 11, and 11 crossovers, respectively.

To test the possibility that reciprocal COs are affected by this uneven distribution of SNPs, we mapped recombination points on the X chromosome from the parental females in Fig. 3b and classified them relative to the SNP dense regions, as shown in Fig. 4. SNPs were assigned parental origin and used to infer haplotype structure along the X chromosome (Methods). Each row corresponds to an individual recombinant fly, ordered by locomotor activity, with parental haplotype blocks shown along the chromosome. The 5 SNP dense regions identified in Fig. 3b are indicated in black at the top of the figure, labeled A–E. Figure 4 indicates the importance of SNP dense regions C and D to the phenotypic differences in the recombinant flies, and that reduced CO frequency and randomness of COs both contribute to the lower than expected resolution of our genetic mapping study above.

### Reduced CO frequency within SNP dense regions

Large regions of unbroken parentage, or haplotype blocks, are apparent in the SNP dense regions, but not in the SNP sparse regions (Fig. 4). The latter is at least partly due to the difficulty of determining parentage in the highly similar SNP sparse regions. The finding of these long haplotype blocks is consistent with failure to better resolve the associating regions as many recombinant flies retained large segments derived entirely from either the *DD-Hi* or *DD-Lo* parental chromosome. Note that these haplotype blocks are on a faint background of the apparently opposite parentage. We cannot distinguish whether this background represents non-CO conversion events, vs sequence errors/ambiguity resulting from the marginal sequence coverage from this single recombinant fly sequencing.

Directly counting COs in the SNP sparse region is not possible since we can’t discern haplotype parentage blocks due to the near-identity of parental sequences. Instead, we infer that at least one CO has occurred due to change in parentage in the SNP dense regions on either side of the SNP sparse region. The observed rate of inferred COs will be an absolute minimum, since we can’t detect COs if an even number of COs occurs, and our estimated frequency will be reduced if there is selection for the same parentage in the adjoining regions SNP dense regions C and D, as we demonstrate below.

**Fig. 5.**
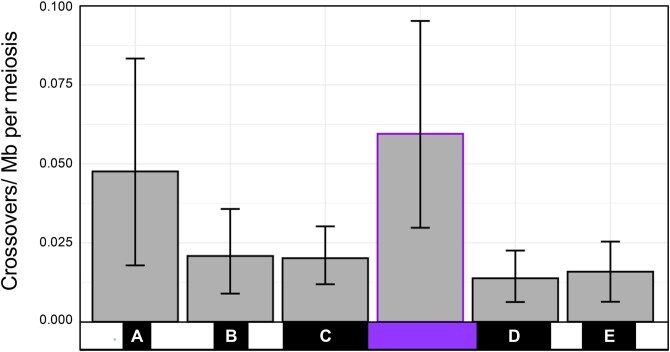
Recombination frequency within SNP dense regions is lower than in adjoining SNP sparse regions. The recombination frequency of each region was calculated for a single meiosis (see Methods). Crossovers per Mb was plotted with 95% confidence intervals generated using bootstrap resampling (*n* = 10,000). SNP dense regions are denoted by black boxes that correspond to the length of each region; the smallest SNP sparse region used for comparison is denoted by the purple box.

Even with this minimum estimate, we observe a recombination frequency that is 3.4-fold higher in this SNP sparse region than in the adjacent SNP dense regions C and D (Fig. 5). Since we are estimating recombination frequency with a small number of COs, a bootstrap resampling was used to estimate confidence intervals for each region (see Methods). This analysis showed that SNP dense regions generally have lower recombination rates compared to the adjacent SNP sparse region than expected by random chance. To contextualize this analysis further, an exact Poisson rate test confirmed the observed 3.4-fold suppression in SNP dense regions C and D is statistically significant for each region individually and sufficient to explain the ∼5-fold lower resolution we obtained with our mapping study (Methods; region C: *P* = 0.009, region D: *P* = 0.001).

### SNPs in regions C and D associate with activity levels in the recombinant flies

**Fig. 6.**
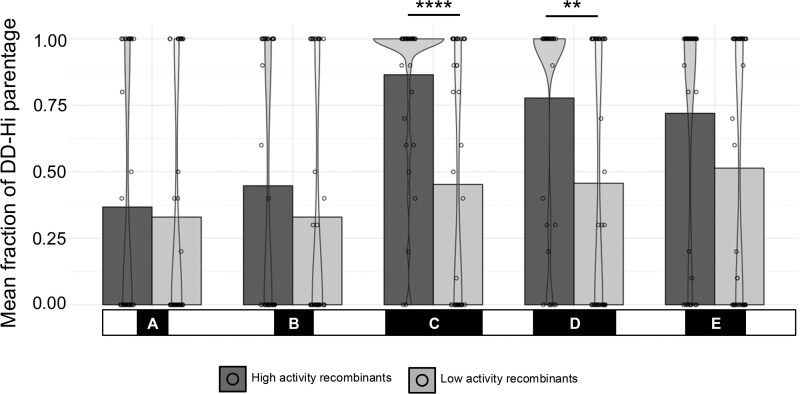
Locomotor activity of each recombinant line plotted as a function of the ratio of *DD-Hi* parentage within each SNP dense region. The percentage of *DD-Hi* parentage was estimated within each SNP dense region for each recombinant fly by counting a full *DD-Hi* tract as 1 and a full *DD-Lo* tract as 0; recombinant flies that contained a crossover event were estimated as a fraction of *DD-Hi* parentage. Data presented as mean with the distribution of individual data points shown by the overlaying violin plot. Asterisks denote significant differences between the high and low activity recombinants within a given region where **P < 0.01 and ****P < 0.0001 (Wilcoxon Rank Sum Test—Bonferroni Corrected, *n* = 40 high activity and 44 low activity).

Our genetic mapping study identifies SNP dense regions C and part of D as containing single nucleotide differences associating with activity levels in the recombinant flies (Fig. 3b). Casual observation of the data in Fig. 4 appears to show much higher *DD-Hi* haplotype blocks in the high activity recombinants, and *DD-Lo* predominating in the low activity recombinants, especially in SNP dense regions C and D. We quantified these parentage distributions in Fig. 6, showing fractional *DD-Hi* parentage for each fly. The distribution of individual recombinants is shown with a violin plot, since the majority of the data are either 0 (full *DD-Lo*) or 1 (full *DD-Hi)*. Parentage of the SNP dense regions that overlap the regions of high association show *DD-Hi* vs *DD-Lo* parentage that strongly correlates with activity levels of the recombinant flies, reaching significance in the 13.9–16.5 Mb region C. The correlation of parentage in the 16.9–18.8 Mb region D is similarly biased, but not nearly as significant as region C, presumably since only part of this region shows significant association. These observations are consistent with the importance of these associating regions in controlling the locomotor activity phenotype.

### Uniform recombination mapping in earliest studies

**Fig. 7.**
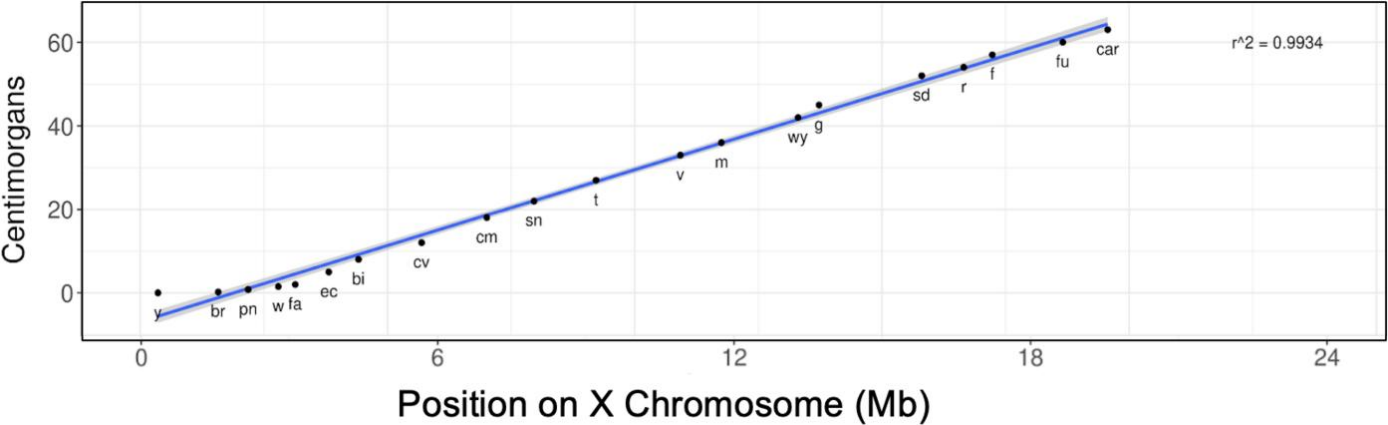
Recombination mapping of classical alleles. Recombination mapping is highly accurate across the central region of the X chromosome when assaying classical alleles on the X chromosome. Each point represents a visible gene, mapped by both classical and molecular techniques. Recombination map positions and base positions on X chromosome are from https://FlyBase.org. The datapoints map to a line with *r*^2^ = 0.9974, plotting map position in centimorgans vs molecular position on the X chromosome.

As summarized above, a number of studies show variation in recombination rates across the central region of the *Drosophila* X chromosome. Given the results we present here, we suspect that these studies could have been influenced by variable distributions of SNPs across the chromosomes. As such, we wondered whether the earliest mapping studies, all of which apparently used the same fly background, would be free from these mapping influences from distinct fly backgrounds. We took the X chromosome mapping data from the study by Morgan et al. (1925) and plotted the given map position in Centimorgans vs the molecular position of the respective genes in flybase.org. The original mapping data was only corrected for frequencies of double recombinants. As shown in Fig. 7, the genes map to a line with an *r*^2^ value of >0.99. Thus, these original map data are incredibly precise, strongly suggesting that later studies were inadvertently affected by inhomogeneous SNPs between the lines used in the crosses.

## Discussion

DA is a critical regulator of arousal and locomotor activity in *Drosophila*. Flies lacking brain DA exhibit the expected phenotype of diminished locomotor activity (Cichewicz et al. 2017). Remarkably, in this study, we identify a sub-line of DA-deficient flies that recover normal levels of locomotor activity, despite the complete absence of brain DA. Using a bulk segregant approach and WGS, we mapped the genetic basis of this recovery to a ∼3.5 Mb region of the X chromosome, with ∼5× lower resolution than expected. We thus changed our focus from precise mapping of this element, to instead, understanding why our mapping resolution was so much lower than expected. We found that mapping was hindered by an uneven distribution of single nucleotide differences within the starting X chromosomes, biasing recombination events and thus limiting our resolution.

While our mapping focused on the X chromosome, we also detected minor associations with the high activity phenotype on the second chromosome. However, due to the nature of our sequencing approach, which used single males with diploid autosomes, we were unable to distinguish individual autosomes. This limitation made it difficult to confidently track inheritance patterns or pinpoint COs outside of the X chromosome. Given these constraints, we concentrated our analysis where we had the highest resolution.

The X chromosomes used in our recombination study display an unusual pattern of sequence divergence, with regions of near-homozygosity, 0.04% divergence, interspersed with 5 distinct ∼1–3 Mb regions with 0.25% average divergence. Our mapping identifies 2 of these SNP-dense regions, C and D (Fig. 3), as associated with the high locomotor activity phenotype. However, the numbers of recombination events in these regions are lower than anticipated, at least partly accounting for the ∼5-fold lower resolution in our mapping vs the anticipated value. Counts of reciprocal COs confirmed a 3.4-fold reduction in CO frequency relative to a nearby region of near homology, suggesting a novel mechanism limiting recombination in these SNP-dense regions. We note the possibility that non-random matings during the 15 generations during which free recombination occurred could have reduced the opportunity for effective recombination (Korol et al. 2000). However, our finding that the recombination frequency in the SNP-sparse region between the SNP-dense blocks C and D is at the expected frequency makes this unlikely.

Previous evidence in *Drosophila* shows variation in recombination rates across the *Drosophila* genome (Chan et al. 2012), particularly near the centromeric and telomeric regions where COs are strongly reduced (Lindsley and Sandler 1977; Comeron et al. 2012; Hughes et al. 2018). Lindsley and Sandler (1977), show variation in the level of exchange across the X chromosome, but less extreme variation than on the autosomes. We note that the hot/cold spots seen by (Chan et al. 2012; Comeron et al. 2012) show variability between each study, and even between specific crosses within a given study (Comeron et al. 2012). We suggest that this variability could be related to patterns of varying blocks SNP density between starting strains as we have seen in our study. The exception to this variation in recombination frequency across the X chromosome is seen in the earliest mapping studies from Morgan's lab (Morgan et al. 1925). These early studies occurred at a time prior to the appearance of discrete background strains of *Drosophila,* which would presumably have differential patterns of SNPs.

We note that the SNP dense region C in our current data is less informative in our genetic mapping than we would have hoped. Figure 4 shows that high and low activity recombinants can have parentage mismatches from *DD-Hi* and *DD-Lo* on either side of these COs. These findings could be interpreted as showing that a large region is required to control activity levels. But this interpretation is confounded by the possibility of non-CO gene conversion events that are beyond the detection level of our sequencing. This could explain our unexpected high activity recombinants that contain region C and D with total parentage from the *DD-Lo* parents, and vice versa.

Where is the genetic diversity in our starting lines coming from? The high and low activity DA deficient lines were isolated after we performed single pair matings of our DA deficient flies (Cichewicz et al. 2017) to our laboratory stock of *w^1118^*. We performed these crosses in an attempt to reduce genetic diversity in our DA deficient flies, but we suspect that instead we added genetic diversity. Our working stock of *w^1118^* has been maintained for decades, while at the same time we maintained a number of *w^1118^* stocks in diverse genetic backgrounds in our laboratory stock collection. Notes contemporaneous to when the crosses were performed indicate that our stock collection contained *w^1118^* in backgrounds including Canton-S, Oregon-R, Berlin, as well as *w^1118^* lines obtained from several colleagues around the world. When these lines were assayed for locomotor activity, they showed a 2–3-fold variation in locomotor activity (data not shown). We thus suspect that sometime in the past decades that our working stock of *w^1118^* was inadvertently contaminated with at least some of these genetic backgrounds. This hypothesis would help to explain the near identity along the majority X chromosomes when comparing the *DD-Lo* and *DD-Hi* flies, since they would have been intermating prior to our single pair matings. The reduced CO frequency in the punctuated regions of SNP density would thus serve to preserve these regions intact, consistent with our observations.

We find no direct precedent in *Drosophila* for our finding that reciprocal COs are selectively suppressed in chromosomal regions with enhanced divergence, though there are relevant examples in mitotic recombination and in other organisms. (Rutherford and Carpenter 1988) examined isogenized X chromosomes for differences in meiotic CO frequencies relative to control chromosomes and found no differences, but the level of divergence in their control chromosomes was unknown. More recent studies show that pre-CO enzymes bind less effectively to diverged regions during mitosis. In *Drosophila* cultured cells, bloom helicase binding and mitotic recombination are impeded by sequence divergence less than 0.5% (Kappeler et al. 2008), similar to the levels in our SNP dense regions. In mouse, divergence as high as 1.8% is not sufficient to trigger heteroduplex rejection during meiosis (Peterson et al. 2020). In yeast, it has long been appreciated that there is a reduced rate of meiotic recombination in heterozygous regions (Borts and Haber 1987), and there is less binding of the mismatch repair protein Msh5 to regions of higher divergence (Dash et al. 2024).

Our findings underscore the importance of chromosomal relatedness when designing genetic mapping studies. Regions of punctuated diversity, like those identified in this study, can significantly skew recombination patterns, leading to biased mapping outcomes. This insight is critical for the broader field of genetic mapping in *Drosophila* and other organisms where similar recombination biases might occur unnoticed.

## Data Availability

Raw WGS data are deposited in the NCBI SRA (http://www.ncbi.nlm.nih.gov/sra) as a BioProject under accession number PRJNA1393392. Fly stocks generated and used in this study will be made available to the research community upon reasonable request.
